# Case of Amputation

**Published:** 1861-08

**Authors:** L. B. Brown

**Affiliations:** Iroquois, Ill.


### CASE OF AMPUTATION.

Henry Euslin, a resident of Iroquois, Ill., aged 66 years, and of scrofulous habit, received an injury of the left ankle joint jumping from a carriage. The foot and ankle become swollen and painful. Domestic remedies were used for nearly two months, when a large sloughing ulcer presented at the heel. A physician was now called and constitutional treatment brought to bear on the sore heel, but without relief.

I visited the patient for the first time in February, 1861. I found him emaciated, having diarrhœa, and suffering considerable pain. The foot and ankle were studded with fistulous openings discharging offensive matter; the tendoachillis, muscles of the heel, and posterior ligaments of the ankle joint were completely destroyed, leaving the os-calcis, tibia and fibula exposed. I spoke of amputation as the only chance for relief, but did not recommend it, owing to the exhausted condition of the patient.

March 2—Visited the patient in company with Drs. Barry, Fowler and Dunn. After stating to the patient and family, the certainty of death without, and the chance of recovery with, an operation, consent was given and the operation performed. Chloroform was given as an anæsthetic, and the limb amputated in its upper third just below the tuberosity of the tibia. The usual dressings were applied, and brandy and opium given during the remainder of the day and night.

March 5—The patient has rested very well since the operation; no pain in the stump; pulse 80; the flaps of the wound lying against each other without any adhesion, or evidence of inflammation. Ordered, in addition to brandy and opium, quinine and beef-tea.

March 6—Pulse 80; suppuration of the stump; discharging unhealthy pus. Ordered the stump washed with tinct-myrrh and covered with pulv. charcoal.

March 9—Patient exhibits signs of exhaustion; the surface covered with cold perspiration, and the wound discharging a large amount of offensive matter; the extremity of the tibia is exposed. Continue the same treatment, with increase of brandy.

March 12—Patient thirsty, skin hot, pulse 90; the stump inflamed and painful for the first time since the operation.

March 15—The discharge from the stump is considerably diminished, and there is some appearance of healthy granulation. Continue the same local and constitutional treatment as before.

April 15—The daily notes of the case since March 15th contain nothing of interest or importance. The wound, owing to the impaired condition of the system, healed slowly. The patient is improving in general health, and the wound is nearly cicatrised.

The above case, I think, is one of interest, considering the advanced age and extreme debility of the patient at the time of the operation. I do not offer it, however, as an anomaly; but, from the fact that I have been censured (for operating) by a few meddlesome people, some of whom claim to belong to the Medical Profession.

L. B. BROWN, M. D., Iroquois, Ill.

